# Hormesis Is Biology, Not Religion

**DOI:** 10.1289/ehp.114-1764167

**Published:** 2006-12

**Authors:** Ralph R. Cook, Edward J. Calabrese

**Affiliations:** RRC Consulting, LLC, Midland, Michigan, E-mail: ralphrcook@chartermi.net; School of Public Health, Environmental Health Sciences Program, University of Massachusetts, Amherst, Massachusetts, E-mail: edwardc@schoolph.umass.edu

Should hormesis, as Thayer et al. (2006) implied in the title of their letter in the November 2006 issue of *Environmental Health Perspectives*, be dismissed by scientists, regulators, and others as simply a new faith-based religion? No. Hormesis is a data-based biological reality, one that challenges the low-dose assumptions that currently drive risk assessment processes used by regulatory and public health agencies worldwide.

As we discussed in our recent commentary (Cook and Calabrese 2006), we believe that default assumptions, however well intentioned, should not trump data in the formulation of public health policy. Published scientific information supporting the hormetic nonmonotonic dose–response curve is extensive. The most recent comes from an article based on a large National Cancer Institute antitumor drug screening database (Calabrese et al. 2006), which reports that effects at low-level exposures are inconsistent with the threshold model and supportive of the hormetic model.

We believe the current regulatory mandated approach of narrowly gathering effect data at high doses of exposure and then dogmatically imputing an excess burden of harmful outcomes monotonically down to and below the markedly lower levels that actually occur in the environment is wrong. This approach is wrong because it censors the observations that can be considered (only high-dose adverse effects and often just the worst-case sentinel effect) and requires the use of nonscientific assumptions that are either untested or untestable. The hormetic model addresses both of those shortcomings. It encourages the collection of data across a broader range of dose and thereby allows evaluation of both risks and benefits (specific and holistic) that would occur at these lower levels. In addition, findings based on the hormesis model are subject to tests using empirical data.

Without evidence, Thayer et al. (2006) argued that we were wrong to suggest that public health might be better served by setting exposure standards at levels using data collected based on the hormetic model. We strongly disagree. With the additional information, we believe policies could be developed that would not only prevent excess disease or death over background but also promote better health, quite possibly for both the general public and more sensitive subgroups.

Although we differ with Thayer et al. (2006) on a number of points, we all seem to agree that hormesis exists. Building on that consensus, perhaps we all can also agree with the perspective recently presented by Rietjens and Alink (2006): the discipline of toxicology should refocus its efforts to better address the regulatory issues of low-dose effects and risk–benefit analysis.

## References

[b1-ehp0114-a0688a] Calabrese EJ Staudenmayer JW Stanek EJ III Hoffman GR. 2006. Hormesis outperforms threshold model in NCI anti-tumor drug screening database. Toxicol Sci 10.1093/toxsci/kfl098 [Online 1 September 2006]. 10.1093/toxsci/kfl098 16950854

[b2-ehp0114-a0688a] Cook RR, Calabrese EJ (2006). The importance of hormesis to public health. Environ Health Perspect.

[b3-ehp0114-a0688a] Rietjens IMCM, Alink GM (2006). Future of toxicology—low-dose toxicology and risk–benefit analysis. Chem Res Toxicol.

[b4-ehp0114-a0688a] Thayer KA, Melnick R, Burns K, Davis D, Huff J (2006). Hormesis: a new religion? [Letter]. Environ Health Perspect.

